# The Role of Genomics in Advancing and Standardising Bacteriophage Therapy

**DOI:** 10.3390/antibiotics15010055

**Published:** 2026-01-04

**Authors:** Narina Abdraimova, Egor Shitikov, Maria Kornienko

**Affiliations:** Lopukhin Federal Research and Clinical Center of Physical-Chemical Medicine of Federal Medical Biological Agency, Malaya Pirogovskaya 1a, 119435 Moscow, Russia; eshitikov@rcpcm.org (E.S.); kornienkomariya@gmail.com (M.K.)

**Keywords:** bacteriophage, phage therapy, genomics, whole-genome sequencing (WGS), antimicrobial resistance (AMR), personalised medicine

## Abstract

Bacteriophage therapy, which employs bacterial viruses to selectively eliminate pathogenic bacteria, has re-emerged as a promising strategy in the face of increasing antimicrobial resistance. However, its widespread clinical implementation is constrained by concerns regarding safety, standardisation, and predictable efficacy. In this review, we examine the key role of genomics in transforming phage therapy from an empirical practice into a standardised and personalised modality of contemporary medicine. We describe how whole-genome sequencing (WGS) provides a basis for safety assessment by enabling systematic screening to exclude virulence factors, antibiotic resistance genes, and markers of lysogeny. WGS also facilitates the prediction of therapeutic efficacy and supports more rational phage selection by identifying receptor-binding proteins and characterising bacterial defence systems. In clinical settings, WGS data are increasingly used to monitor the evolution of bacterial populations and to adapt phage cocktails during treatment, thereby supporting personalised, adaptive phage therapy. Looking ahead, further progress is likely to come from integrating synthetic biology and artificial intelligence to engineer phage-based therapeutics with programmable specificity and predictable properties. Together, these developments are shaping a new paradigm of phage therapy as a scientifically grounded, standardised and controlled strategy to treat infections caused by antibiotic-resistant bacteria.

## 1. Introduction

Antimicrobial resistance (AMR) remains one of the major global threats to public health. The spread of antibiotic-resistant pathogens not only undermines the efficacy of conventional antibiotic therapy but also imposes a substantial economic burden. Each year, infections caused by such microorganisms are responsible for millions of deaths [1,2]. This situation is further aggravated by the slow pace of antibiotic development, making the search for alternative approaches to treating bacterial infections an urgent priority.

Bacteriophage (phage) therapy is one of the most promising strategies to address the AMR crisis. In contrast to antibiotics, bacteriophages offer several unique advantages [3]. Their high specificity minimises disruption of the commensal microbiota, while their ability to replicate at the site of infection supports high local concentrations of the therapeutic agent. Their distinct mechanism of action underlies their activity against antibiotic-resistant strains. Phages can also be effective against biofilm-associated infections, as they produce enzymes that degrade the extracellular matrix [4,5]. The relative ease of isolating new bacteriophages and scaling up their production further increases the attractiveness of this approach.

Historically, phage therapy was introduced long before the advent of modern molecular methods. The first clinical applications were reported as early as 1918–1919 [6,7]. At that time, the selection of therapeutic agents relied exclusively on their ability to lyse target bacteria, assessed primarily by two phenotypic methods: in liquid culture (the Appelmans method), based on broth clearing, and on solid media (the Gratia method), based on the formation of lysis zones (plaques) [8,9]. Although useful, these approaches did not reveal the molecular mechanisms underlying efficacy or the development of resistance, which hindered standardisation of therapy and contributed to contradictory clinical data. Consequently, the introduction of antibiotics with predictable efficacy and a broad spectrum of activity in the 1940s led to the near-complete abandonment of phage therapy for several decades [10].

The renewed interest in phage therapy in the 2000s, driven by the escalating prevalence of antibiotic resistance, occurred in parallel with the rapid progress in genomic technologies. Whole-genome sequencing (WGS) enabled a shift from purely phenotypic selection to systematic molecular analysis, providing comprehensive insight into genome organisation and the mechanisms that govern phage-bacterium interactions. In the context of phage therapy, one of the key contributions of WGS has been the establishment of safety criteria. Genomic analysis allows for the evaluation of phages across several critical parameters, such as confirmation of a strictly lytic life cycle and exclusion of virulence and antibiotic resistance genes, as well as taxonomic placement based on currently accepted frameworks. This is particularly important because morphologically similar phages can possess distinct genetic repertoires that determine their safety profiles [5,11]. As genomic approaches have developed, their application has expanded from safety assessment to the prediction of efficacy and the personalisation of therapy. As a result, WGS has made the selection of therapeutic phages more transparent and reproducible, providing a basis for the rational design of phage cocktails and their adaptation during treatment.

In this review, we examine the pivotal role of genomic technologies in the development of modern phage therapy, with a particular focus on how whole-genome sequencing can be translated into a practical framework for therapeutic decision-making. Tracing the evolution of phage genomics from its origins to clinical integration, we highlight how genomic data bridge experimental characterisation with clinical applicability. The article summarises genomic analysis pipelines that underlie the selection of safe therapeutic phages and places particular emphasis on the prediction of phage efficacy through the analysis of phage-host interactions. It also discusses the use of WGS to personalise treatment, monitor in vivo pathogen evolution, and adapt phage cocktails during therapy. Finally, we outline future prospects for applying artificial intelligence and synthetic biology to the rational design of phages and to the development of a clinically standardised, controllable strategy for combating antibiotic-resistant infections.

## 2. Phage Genomics in Historical Perspective

**Figure 1:**
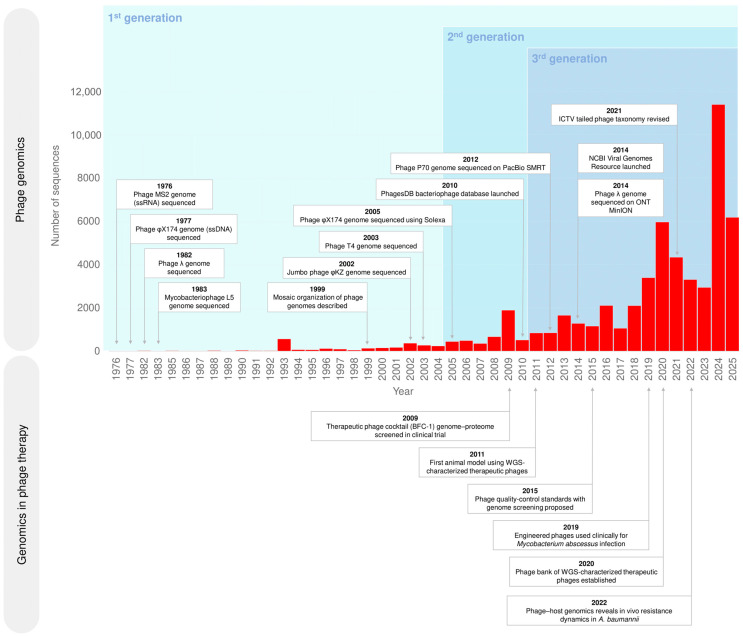
Timeline of major milestones in the development of bacteriophage genomics (upper part of the diagram) and bacteriophage genomics in the context of therapy (lower part). The bar chart shows the number of phage genomes sequenced per year from 1976 to 2025 (Data source: NCBI (National Center for Biotechnology Information) Virus, https://www.ncbi.nlm.nih.gov/labs/virus/vssi/#/; accessed on 27 November 2025).

Advances in sequencing technologies have enabled the systematic accumulation of complete bacteriophage genome sequences. As illustrated in Figure 1, this expansion now extends well beyond basic research and directly contributes to the development of specialised databases, the revision of taxonomic frameworks, and the establishment of standards for therapeutic phage use.

The pioneering sequencing of the bacteriophages MS2 (ssRNA) [12] and φX174 (ssDNA) [13] in the 1970s marked the beginning of a new era in the systematic study of phage genome organisation. This methodological framework was soon applied to other key model phages, including λ [14], T4 [15], L5 [16], and later to giant jumbo phages [17]. Comparative analysis of the growing collection of sequences led to one of the most important early insights: the recognition of the mosaic structure of phage genomes, in which genetic material is organised into modules derived from diverse sources [18]. This discovery fundamentally changed our understanding of bacteriophage evolution, showing that phage genomes are highly dynamic, extensively shaped by recombination, and actively involved in horizontal gene transfer (HGT).

A new stage in phage genomics began with the emergence of high-throughput next-generation sequencing (NGS), which greatly increased data output and reduced costs compared with Sanger sequencing [19]. The first application of the sequencing-by-synthesis (Solexa) platform to resequence bacteriophage φX174, published in 2005, represented a pivotal advance [20]. Due to its efficiency and accessibility, NGS became the main driver of genome data accumulation and made WGS a routine component of studies on newly isolated phages.

Third-generation, long-read sequencing technologies further expanded the possibilities for assembling complete phage genomes. An early demonstration of their practical value was provided in 2012 with the assembly of the *Listeria monocytogenes* phage P70 genome, in which PacBio SMRT reads were successfully combined with Sanger data [21]. In 2014, the potential of long-read sequencing was reinforced by the generation of a reference genome for phage λ using the MinION platform (Oxford Nanopore), consolidating the role of these technologies in resolving complex regions of phage genomes [22].

The rapid accumulation of phage genomes has led to the creation of specialised repositories for their systematic organisation. One of the earliest initiatives was PhagesDB, which provides centralised access to genome sequences and associated metadata [23]. This area has been further advanced by the development of the large-scale NCBI (National Center for Biotechnology Information) Virus platform, which integrates data on viruses, including bacteriophages, within a single standardised resource [24].

To support the analysis of these expanding genomic datasets, a wide range of software tools has been developed for automated phage genome processing. For the primary annotation, specialised phage pipelines such as Pharokka [25] and PHANOTATE [26] are now widely used. In parallel, tools for taxonomic and comparative genomic analysis have been developed [27,28], along with early approaches for in silico prediction of bacterial hosts from phage sequences [29]. More comprehensive platforms have since emerged that integrate taxonomic, phylogenetic, and structural analyses [30,31,32,33]. A distinct subfield focuses on prophages, defined as integrated phages within bacterial genomes. For these tasks, search and prediction tools such as PHAST [34] and its improved versions, PHASTER and PHASTEST [35,36], are employed. These tools facilitate the study of prophage diversity and functional potential and improve the accuracy of genome data interpretation.

The expansion of data infrastructure and the accumulation of genome sequences have also prompted a revision of fundamental concepts in phage biology, particularly of taxonomy. Historically, bacteriophage classification relied on a limited set of characteristics, including host range, nucleic acid type, and life cycle features [37,38,39,40]. The advent of electron microscopy enabled the development of a morphology-based classification system, which was formally adopted by the ICTV in 1981 with the recognition of three major families of tailed phages: *Myoviridae*, *Siphoviridae* and *Podoviridae*. However, comparative genomics revealed that phylogenetic trees inferred from sequence data often did not align with this morphology-based classification, which led to the abandonment of morphology as the primary taxonomic criterion [41]. This culminated in a major revision of ICTV taxonomy in 2021–2022, when a new system based on genome architecture and evolutionary relatedness was introduced [42]. This approach improves the biological robustness of taxonomic units and facilitates comparison of phages across diverse research and therapeutic collections.

These technological advances created the basis for integrating genomics into phage therapy. As complete genomes of phages active against clinically relevant pathogens became increasingly available [17,43,44,45,46,47,48,49,50,51,52], the first examples of their therapeutic use were reported. In 2009, a preliminary genomic and proteomic safety assessment of the BFC-1 cocktail targeting *Pseudomonas aeruginosa* and *Staphylococcus aureus* was carried out, followed by its clinical application [53]. In 2011, the first animal study using fully annotated phages to treat a pulmonary infection caused by *P. aeruginosa* was published [54].

Growing interest in the clinical phage use led to the publication in 2015 of the first recommendations for evaluating their safety and efficacy, in which genome-based screening was formally established as a standard component [55]. At the same time, the use of genomics expanded during clinical implementation of phage therapy. In several cases, WGS has been employed not only to select active phages but also to monitor phage and bacterial populations during treatment [56,57,58,59]. These studies highlighted the importance of a dynamic perspective that recognises the capacity of phages and bacteria to co-evolve under selective pressure.

The extensive use of WGS in phage selection and therapeutic monitoring has increased the need for standardisation and for ready access to well-characterised phages, which has driven the establishment of specialised phage biobanks. Numerous countries now maintain such resources. For example, the Israeli Phage Bank [60] contains a collection of more than 300 phages selected for their therapeutic relevance, and WGS is prioritised for isolates in the highest clinical demand. In Belgium, within the “magistral phage medicine” framework for personalised phage therapy, a national phage bank has been created at Queen Astrid Military Hospital [61]. Phages in this collection undergo sequencing and bioinformatic screening to confirm the absence of toxin genes, antibiotic resistance determinants, and markers of lysogeny. In the United States, the clinically oriented PhageBank [62] operates under conditions in which complete genome sequences are mandatory for phages used in IND (investigational new drug) protocols. In Australia, the national Phage Australia initiative maintains a centralised biobank [63]. Deposition of a phage into this collection requires confirmation of preparation purity, including the determination of its genome sequence. Long-established therapeutic phage collections at the Eliava Institute in Georgia and the Hirszfeld Institute in Poland have also made major contributions [64,65]. In these collections, WGS is performed selectively for clinically and scientifically prioritised isolates and is not yet mandatory for all deposits.

Alongside creation of formal biobanks, substantial phage collections have been developed within academic and commercial programmes. These collections often exceed the size of dedicated banks and focus on specific pathogens. A notable example is the SEA-PHAGES programme (University of Pittsburgh), which has isolated over 25,000 phages, predominantly against non-pathogenic soil mycobacteria. However, some of these phages have shown activity against related pathogenic species and have been used therapeutically [66]. Another example is the Bundeswehr Institute of Microbiology (Germany), which has assembled extensive panels of phages targeting multidrug-resistant (MDR) *K. pneumoniae* strains, with their characterisation strongly supported by WGS in research projects [67].

Together, these specialised phage banks and broader collections form a key interface between genomics and clinical practice. They facilitate the selection of therapeutically relevant phages and provide essential infrastructure for personalised phage therapy.

## 3. Genomic Analysis as the Basis for Selecting Safe Therapeutic Phages

Safety is a central requirement for the clinical use of bacteriophages. At present, regulatory documents, including EMA and FDA guidelines as well as national standards in countries with established phage therapy practice, describe requirements for genetic safety assessment largely in general terms [68]. In parallel, the literature presents a wide range of additional approaches for interpreting phage WGS data to identify and exclude virulence determinants, resistance genes and mobile genetic elements (MGEs). Table 1 summarises an extended framework and illustrates how WGS data can be used to support standardised assessment of phage safety and suitability for therapeutic application.

The first practical step in this workflow is stringent quality control of genome assemblies. At this stage, assemblies reconstructed from sequencing reads are examined for completeness and for the absence of artefacts. Incomplete assemblies can lead to fragmented or ambiguous gene predictions, while contamination with foreign DNA (bacterial or phage) may result in false-positive detection of undesirable genes, including toxin determinants or antibiotic resistance genes (ARGs). Specialised tools such as CheckV [75], VirSorter2 [76] and VIBRANT [77] are used to evaluate the completeness and “purity” of phage genomes.

After quality control, genome annotation is performed. In this step, genes are identified and their putative functions are predicted. The resulting structural and functional map provides the basis for subsequent targeted safety assessments. Phage-specific annotation pipelines such as Pharokka [25], PHANOTATE [26] and Sphae [78] are commonly used for this purpose.

The next step is to determine the phage life cycle. Only strictly lytic phages, which cause productive lysis of the host cell without integrating into the bacterial chromosome, are considered suitable for clinical use [79,80]. Temperate phages capable of lysogeny pose a risk of HGT and may increase bacterial virulence. Genome-based screening allows the detection or exclusion of lysogeny-associated markers, including integrase, repressor and excisionase genes, as well as attP/attB attachment sites [81,82]. In silico life-cycle prediction tools such as PhaTYP [69] and BACPHLIP [70] are now widely used to minimise the inclusion of temperate phages in therapeutic cocktails.

However, caution is required, as the presence of an integrase gene does not necessarily imply a lysogenic phenotype in standard laboratory infection assays (e.g., plaque morphology and liquid-culture tests). For example, the *S. aureus* phage SauPS-28 encodes a site-specific integrase yet displays a lytic phenotype in plaque assays, forming clear plaques without signs of turbid centres that might indicate lysogeny. This suggests that the integrase gene may be non-functional or that essential host factors required for integration are absent [83]. The conditional nature of lysogeny is further illustrated by the well-studied bacteriophage λ, where successful integration depends not only on the phage-encoded integrase but also on host-encoded architectural factors (notably Integration Host Factor, IHF) [84], highlighting that integrase-like sequences alone are imperfect predictors. A well-known example of the opposite scenario is the *Vibrio cholerae* phage CTXφ, which integrates into the host genome via host-encoded recombinases and therefore lacks a phage-encoded integrase [85]. Taken together, these examples illustrate that both false-positive and false-negative genomic signals can occur. Therefore, therapeutic development applies a conservative decision rule in which candidates carrying lysogeny-associated genes are excluded even if lysogeny is not detected experimentally, while candidates lacking canonical markers still require careful evaluation to exclude non-canonical integration routes.

A further critical step is to screen phage genomes for virulence factors (particularly toxins) and antibiotic resistance genes (ARGs). Although classical bacterial toxins (such as diphtheria, Shiga, and cholera toxins) are traditionally associated with temperate phages [86,87,88,89], their potential presence cannot be ruled out based on life-cycle prediction alone. The systematic exclusion of ARGs is equally critical. Despite the fact that the great majority of known phage genomes, including well-characterised lytic phages, do not contain ARGs [47,90,91,92], recent data suggest that their prevalence may be underestimated. For example, Wang et al. [93] reported functionally active trimethoprim-resistance dihydrofolate reductase (*dfrA*) genes in lytic phage genomes and showed that the *dfrA* gene carried by the lytic *E. coli* phage vB_EcoM_BMB16 is expressed during infection and confers trimethoprim resistance to its host. In the presence of trimethoprim, such phage-borne *dfrA* genes promote bacterial growth during infection and enhance phage reproduction, indicating that ARGs can be selected even among lytic phages. This evidence makes the rigorous genomic screening for both toxin and resistance genes an indispensable component of phage safety assessment.

In practice, screening for virulence and resistance determinants relies on specialised databases such as the Comprehensive Antibiotic Resistance Database (CARD) and the Virulence Factor Database (VFDB) [71,94], which are integrated into phage-oriented pipelines, including Pharokka [25]. Sphae [78] is also useful, as it combines annotation, life-cycle prediction and targeted filtering of potentially harmful genes (integrases, toxins, MGEs), which is particularly valuable in a therapeutic context.

Particular attention during annotation should be given to hypothetical proteins, which often account for more than half of the predicted proteome. This “dark matter” of the phage proteome presents a biosafety concern, as unannotated sequences may encode toxins or regulatory and metabolic proteins. To mitigate these risks, more in-depth functional characterisation is required, including structural modelling, conserved domain analysis and homology searches against databases such as InterPro [72], Pfam [73] and HHpred [74]. In recent years, machine-learning-based approaches for predicting potentially harmful functional motifs have also emerged as a promising direction [95,96]. The broader challenge of annotating hypothetical phage proteins, including strategies for functional assignment, has been comprehensively discussed elsewhere [97].

The final component of comprehensive genome assessment is the determination of the phage’s taxonomic position. Accurate classification not only organises individual findings but also allows properties of new isolates, such as their propensity for lysogeny or toxin transfer potential, to be inferred from data on closely related phages [98]. Tools such as VIRIDIC [30], taxMyPhage [33] and PhageScope [31] are currently used to phages assign phages to taxa on the basis of genome sequences. In parallel, specific PCR assays with taxon-specific primers can be applied for rapid preliminary verification [99,100]. Phylogenetic analysis therefore serves as an important link that integrates individual phages into the broader body of knowledge on their taxonomic group.

Once the genomic safety of a phage has been established, the next key task is to ensure stability and purity during manufacturing. Phages intended for clinical use should exhibit high genetic stability and a low propensity for recombination. To minimise the risk of undesired genomic changes, it is advisable to use well-characterised production strains that lack active prophages and MGEs. Systematic monitoring of phage genome stability during serial passaging is also recommended. Routine genomic quality control before the release of each production batch is essential, as it enables the detection of rearrangements, point mutations or contamination that could compromise the safety or efficacy of the phage product.

## 4. Predicting Phage Efficacy Through Genomic Analysis

Predicting the efficacy of phage therapy remains a major challenge, particularly when compared with the more standardised protocols available for safety assessment. Successful infection depends on a cascade of molecular events, including the recognition of bacterial surface receptors by bacteriophages and evasion or suppression of bacterial defence systems. Genomic analysis offers a way to address this complexity by supporting the development of dual-focus predictive strategies that consider both bacterial susceptibility markers and phage host-range determinants. Although the molecular details of many phage–bacterium interactions remain incompletely understood, accumulated data are already outlining the basis of future predictive models.

A key predictor of successful phage-bacterium interaction is the presence of specific receptors on the bacterial surface and the corresponding receptor-binding proteins (RBPs) on the phage that mediate adsorption [101,102]

Phage RBPs are typically modular, multi-domain proteins located on tail fibres, tail spikes, or baseplate structures, with a conserved N-terminal region responsible for virion attachment and a variable C-terminal domain conferring receptor specificity. This modular organisation enables rapid evolutionary adaptation through point mutations, recombination, and domain exchange, facilitating host-range shifts and receptor switching [101].

In bacteria, phage receptors comprise a diverse set of surface-exposed cell-envelope components. In Gram-positive organisms, reported receptors include secondary cell wall glycopolymers, such as wall teichoic acids (WTAs) and related wall polysaccharides. In addition, phages may recognise lipoteichoic acids, capsular or extracellular polysaccharides, peptidoglycan-associated motifs, and, in some cases, membrane proteins [103,104]. In Gram-negative bacteria, phage receptors are associated primarily with the outer membrane and include lipopolysaccharide (LPS) structures (notably the O-antigen), outer-membrane proteins (e.g., porins, transporters), and capsular polysaccharides (K-antigens) [105,106]. The latter are particularly important in species such as *Klebsiella pneumoniae* and *Campylobacter jejuni*, where capsule diversity strongly constrains phage host range. Beyond these structures, phages can also utilise motility apparatus such as type IV pili and flagella as receptors [107].

Characterisation of phage RBPs and their corresponding bacterial receptors typically involves a combination of genomic, structural, and functional approaches. Initial identification of putative RBPs often relies on in silico screening of phage genomes for genes located in tail fibre, tail spike, or baseplate regions. These candidates are selected based on homology to known RBP families or the presence of domains characteristic of host recognition modules, and their function is subsequently validated experimentally [101]. For bacterial receptors, a common strategy involves isolating spontaneous phage-resistant mutants, followed by WGS to identify mutated loci. The role of candidate receptors is then confirmed through targeted gene knockouts, complementation assays, and adsorption or binding tests [108,109].

The practical value of linking receptor architecture to RBP features is especially clear in encapsulated pathogens. The importance of these mechanisms is particularly evident in *K. pneumoniae*, where the capsule acts as both the primary barrier and the main determinant of phage susceptibility [110]. In this system, the key determinants of phage adsorption are RBPs with depolymerase domains that degrade the capsule and other exopolysaccharide barriers, enabling access to the cell surface. Routine in silico identification of depolymerases is supported by machine-learning tools such as DePP [111], DepoScope [112], and PhageDPO [113]. The potential of an approach based on combined analysis of phage RBPs and bacterial receptors was demonstrated by Boeckaerts and colleagues, who showed that interaction predictions generated with PhageHostLearn for *K. pneumoniae* were successfully validated experimentally [114].

The principles developed for these systems are increasingly being adapted to other pathogens. For *S. aureus*, the PhARIS tool was developed to automatically identify RBPs from phage genomes and to predict their binding specificity [115]. In cases where a single RBP sequence is insufficient for reliable prediction, for example, when local structural elements or individual amino acid substitutions critically alter specificity, it is advantageous to use PHIStruct [116]. This tool converts RBPs extracted from phage genomes into features that reflect their probable three-dimensional structure and binding regions, thereby improving the accuracy of phage–host matching. This is of particular utility for newly isolated phages, whose RBPs show low sequence similarity to previously characterised proteins.

The presence of a suitable receptor is a necessary but not always sufficient condition for productive infection, because the outcome is also shaped by intracellular bacterial defence mechanisms. These include restriction–modification systems, CRISPR–Cas and a wide range of other dedicated anti-phage modules, which can be detected using tools such as DefenseFinder [117] and PADLOC [118]. On the phage side, critical genomic determinants include restriction inhibitors [119], CRISPR suppressors (Acr proteins) [120], and DNA-modifying enzymes that mask the phage genome [121]. Comprehensive overviews of bacterial anti-phage defence systems and phage counter-defence strategies are available in several recent focused reviews [122,123].

Together, these data show that accurate assessment of phage–bacterium interactions requires consideration of both receptor-level compatibility and potential intracellular defence mechanisms, although the relative contribution of these layers can vary markedly between specific phage–host pairs. For example, in a panel of *Escherichia* strains interaction outcomes could be predicted using genomic data alone, with adsorption-related factors remaining dominant predictors even in integrated models [124]. Similarly, genomic markers reflecting variation in cell wall components and surface molecules have been identified as primary predictors of phage susceptibility in *S. aureus* [125]. At the same time, a recent large-scale genome-wide association study (GWAS) in clinical *S. aureus* isolates identified additional genetic loci significantly associated with resistance, including regulatory and metabolic determinants that influence the expression of surface structures, stress responses and cellular metabolism [126].

Thus, the integration of genomic data from both the target bacterium and candidate phages provides the basis for the rational design of therapeutic cocktails. Combining phages that recognise different receptors and employ distinct mechanisms to overcome anti-phage defences can broaden the spectrum of activity and has been shown to delay or prevent the emergence of resistance. This strategy has been validated experimentally: a combination therapy that included antibiotics and a rationally designed phage cocktail tailored to the receptor profile yielded coverage of at least 96% of 153 *P. aeruginosa* isolates and proved effective in vivo in a wound infection model. The same approach was also successfully applied to *S. aureus* [127].

A logical development of this strategy is the design of ready-to-use, region-specific phage formulations. In a large study by Koncz et al. [128], genomic analysis of *Acinetobacter baumannii* showed that in each region a small, stable set of clones predominates. On the basis of these data, the authors proposed targeted phage cocktails capable of suppressing over 95% of local isolates in both in vitro and in vivo settings, with minimal emergence of resistance.

## 5. Genomics-Driven Personalisation in Phage Therapy

Genomics has increasingly moved beyond routine phage characterisation and is now becoming a practical tool to support evidence-based decision-making during therapy. The core mechanism of this approach is the sequential analysis of pathogen and phage genomes, which makes it possible to track the dynamics of resistance development, monitor the stability of therapeutic agents, and adjust cocktail composition in a timely manner.

A representative example is the study by Liu et al. [57], who applied this strategy to treat a disseminated infection caused by MDR *A. baumannii*. Analysis of isolates collected before and during therapy showed that resistance emerging after 48 h was associated with mutations in the capsule biosynthesis gene cluster, which encodes the primary receptor for the administered phages. Genomic analysis identified the underlying resistance mechanisms and confirmed the genetic stability of the phages throughout treatment. Similarly, in the study by Yang et al. [58], WGS was used to explain reduced phage susceptibility in carbapenem-resistant *P. aeruginosa*. The authors demonstrated that phage resistance was linked to mutations in lipopolysaccharide biosynthesis genes, which serve as phage receptors. A comparable approach was used in the clinical case reported by Blasco et al. [56] describing a prosthetic vascular graft infection caused by *P. aeruginosa*. Comparative genome analysis of sequential isolates obtained before and after phage therapy showed that clinical relapse was associated with the emergence of phage-resistant variants characterised by enhanced biofilm formation and an altered antibiotic susceptibility profile.

It is important to note that in the studies discussed, WGS serves not only to identify resistance mutations but also to assess the physiological trade-offs (fitness costs) that often accompany the development of phage resistance. For example, capsular polysaccharide-deficient mutants of *A. baumannii* exhibit reduced virulence and increased susceptibility to clearance by the host immune system [129,130]. Similarly, phage-resistant variants of *P. aeruginosa*, as demonstrated by Yang et al. [58], are associated with complex phenotypic changes, including shifts in antibiotic resistance, virulence, motility, and biofilm formation capacity—all of which can be directly linked to the identified genomic alterations. However, as noted by Liu et al. [57], these effects are not universal; phage resistance can also lead to pleiotropic consequences, including enhanced biofilm formation (as in the case reported by Blasco et al. [56]) or even increased antibiotic resistance in some scenarios [131,132]. In the long term, the systematic accumulation and analysis of such data should form the basis for predictive models. These models would allow clinicians to not only select effective phages but also account for the likely evolutionary response of bacteria, directing it toward a clinically favourable outcome.

An even deeper understanding of infection dynamics and therapeutic response can be achieved by combining WGS with metagenomic analysis. In the study by Stellfox et al. [59], WGS of *Enterococcus faecium* from blood samples was combined with metagenomic sequencing of stool samples from a patient with recurrent bacteraemia. Genomic data indicated that the patient had been colonised over an extended period by closely related *E. faecium* strains, with invasive isolates likely arising from the intestinal population. Incorporation of phage therapy into the antimicrobial regimen resulted in several months of clinical improvement and a reduction in the intestinal burden of *E. faecium*. However, the subsequent decline in treatment efficacy was attributed not to the emergence of phage resistance, but to the development of a neutralising antibody response against the phages and an increase in the proportion of vancomycin-resistant strains.

Another important application of WGS in phage therapy arises when safe, naturally occurring phages are either unavailable or ineffective against a specific clinical isolate. In such cases, WGS provides the basis for the rational design of personalised phage cocktails. Dedrick et al. [133]. reported the treatment of a chronic, life-threatening infection caused by *Mycobacterium abscessus* in a patient with cystic fibrosis using genetically engineered bacteriophages. On the basis of comprehensive genomic characterisation, the researchers created a personalised cocktail of three phages, two of which were converted from temperate to lytic through targeted genetic modification. This case represented the first clinically validated example of successful therapeutic use of engineered phages. It marked the beginning of a new era in the use of genetically modified phages derived from temperate precursors and has since been supported by several subsequent studies on phage therapy for mycobacterial infections [134,135,136].

## 6. Conclusions and Future Directions

The modern history of phage therapy is closely linked to genomic technologies. Today, the clinical application of any bacteriophage is rarely considered without comprehensive genomic characterisation. Moreover, genomics has moved beyond a purely descriptive role and has become central to standardisation, efficacy prediction and rational design of next-generation therapeutic phage products.

Despite this progress, the growth of the field has also revealed systemic challenges. The most fundamental barrier is the practical feasibility of routine WGS in clinical and resource-limited settings. The costs of sequencing and downstream analysis, along with the required infrastructure, can substantially limit accessibility and increase turnaround time [137]. These limitations are particularly pronounced in low-resource or decentralised healthcare environments [138]. Potential solutions include the development of shared regional sequencing facilities and referral networks, the implementation of standardised, reproducible bioinformatic pipelines, and stepwise diagnostic strategies in which rapid, lower-cost assays support initial therapeutic decisions, while WGS is prioritised for cases where genome-level resolution is essential [139]. Addressing these practical barriers is critical for the timely, scalable, and equitable adoption of phage-based therapeutics [138].

Beyond these infrastructural limitations, a major scientific and regulatory issue is the absence of unified standards for the genomic validation of therapeutic phages. The development of such frameworks is an active area of research [62,140,141,142], and their adoption is likely to represent a pivotal turning point, establishing clear regulatory boundaries and facilitating the broader clinical implementation of phage therapy. In this context, the concept of a “genomic passport” for a phage or phage cocktail is particularly important. Such a passport should include a complete genome annotation, confirmation of a strictly lytic life cycle, evidence for the absence of genes of concern (toxin genes, resistance determinants, integrases), as well as phenotypic testing and stability data. Implementing a genomic passport would ensure origin transparency, batch-to-batch reproducibility, and full traceability for each therapeutic phage strain, thereby providing a reference framework for regulatory procedures.

Another critical challenge is the limited availability of experimental data on lytic efficacy for specific phage–bacterium pairs. Although genomic data are accumulating rapidly, only functionally characterised interactions provide a robust foundation for building accurate machine learning models and for investigating the mechanisms of phage infection using systems biology approaches. Transcriptomic methods already allow the analysis of regulatory networks and bacterial stress responses, the mapping of transcription start sites and the identification of host range determinants [46,143,144,145]. Metabolomics reveals how phages reprogram cellular metabolism, whereas proteomics provides functional validation of these processes, from the characterisation of phage enzymes with therapeutic potential to the monitoring of clinical samples [146,147,148].

The expanding body of systems biology data is enabling a new paradigm: the rational design of therapeutic phages with predefined properties. Modern tools already permit precise genome editing, retargeting tail proteins to alter host range and the introduction of CRISPR–Cas systems for selective targeting of bacterial genes [149,150,151]. Scalable multiplex editing using retron and recombinase-based multiplex genome engineering platforms is expected to accelerate the generation of engineered phage libraries [152]. Technologies such as SMART (split–modify–assemble–reboot) and modular phage genome assembly via Golden Gate Assembly enable the de novo construction of functional phage genomes, including those that are large (40–50 kbp) and have a high G+C content (63–66%) [153,154,155]. Together, these approaches provide a methodological basis for precise editing and engineering of phage genomes, including bacteriophages with predefined properties for personalised therapeutic applications.

At the same time, the development of standardised phage platforms is becoming increasingly important for simplifying the design and regulatory approval of new therapeutic bacteriophages. Rather than engineering each phage de novo, such platforms would consist of unified bacteriophage backbones that have been thoroughly characterised with respect to genetic and biological safety. Their main advantage is that they can be adapted to specific clinical needs, for example, by modifying receptor-recognition modules, without repeating the full preclinical evaluation.

Although most of these approaches are currently at a conceptual or preclinical stage, the growing interest in genetically engineered phages also raises broader ethical, biosafety and public acceptance questions, including how potential off-target effects, horizontal gene transfer and environmental release should be evaluated, and how information on genetic modifications and genomic stability should be communicated [156,157,158]. Engaging with these questions through rigorous, science-based evaluation is therefore a critical component of the field’s maturation. Establishing clear norms for safety and communication will not only address legitimate public concerns but also provide the structured environment in which the therapeutic potential of phage engineering can be rigorously tested and, if validated, responsibly realised.

Taken together, advances in genomics and synthetic biology, along with a growing recognition of the attendant challenges, are driving phage therapy into a new phase. Therapeutic success is becoming defined less by the empirical hunt for active phages and more by the capacity for the systematic design, standardisation, and controlled deployment of therapeutic agents whose properties are specified at the genomic level.

## Figures and Tables

**Table 1:** Genetic safety criteria for bacteriophages considered for therapeutic use.

Estimated Parameter	Genomic Features Assessed	Limitations	Required Criteria	Tools
Life cycle (exclusion of lysogeny)	Markers of lysogeny: integrase, repressor and excisionase genes; attachment (att) sites	Integrases may be non-functional; att sites may be falsely detected; machine-learning (ML)-based classifiers are probabilistic—when predictions conflict with	Strictly lytic phage; no markers of lysogeny	PhaTYP [69], BACPHLIP [70]
Virulence factors	Genes encoding virulence determinants; domain architecture of the encoded proteins	Databases are incomplete; domain mosaicism can produce borderline matches; interpretation requires consideration of genomic context and the full domain composition of the protein	Absence of genes encoding virulence factors	Pharokka [25], VFDB [71]
Antibiotic resistance genes (ARGs)	Full-length ARGs and their operons; functionally relevant domains (e.g., dihydrofolate reductase, DHFR)	High rate of false-positive hits due to conserved domains	Absence of ARGs	Pharokka [25]
High-risk hypothetical proteins	Hypothetical open reading frames (ORFs) with toxin-like or regulatory motifs; structural predictions	Large number of ORFs without homologues; structural predictions may be inaccurate; residual risk of cryptic toxin-like motifs remains	Absence of suspicious high-risk hypothetical ORFs	InterPro [72], Pfam [73], HHpred [74]
Taxonomy	ANI/AAI and intergenomic distances; membership in clades with or without documented molecular risks	Taxonomic assignment serves only as an approximate indicator of risk: there is substantial genetic and functional heterogeneity within taxa, and clade boundaries defined by different tools often do not coincide	Membership in a taxon with no known molecular risk factors	VIRIDIC [30], taxMyPhage [33], PhageScope [31]
Genetic stability	Concordance of WGS data with the reference; absence of single nucleotide polymorphisms (SNPs)/indels in RBPs and lysis modules and of major genomic rearrangements; no evidence of mixed populations	Subclonal variants may be below the detection threshold; cryptic minor clones may persist	Production-batch genome is stable; no rearrangements or contaminations detected	Whole-genome resequencing with read mapping, variant calling and comparative genomics

## Data Availability

No new data were created or analysed in this study.

## Abbreviations

AAI	average amino acid identity
AMR	antimicrobial resistance
ANI	average nucleotide identity
ARGs	antibiotic resistance genes
att	attachment sites (attP/attB)
BACPHLIP	BACterioPHage LIfestyle Predictor
CARD	Comprehensive Antibiotic Resistance Database
CRISPR	clustered regularly interspaced short palindromic repeats
EMA	European Medicines Agency
FDA	Food and Drug Administration
HGT	horizontal gene transfer
ICTV	International Committee on Taxonomy of Viruses
IND	investigational new drug
MDR	multidrug-resistant
MGEs	mobile genetic elements
ML	machine learning
NCBI	National Center for Biotechnology Information
ORFs	open reading frames
RBP	receptor-binding protein
SMART	split–modify–assemble–reboot (phage genome engineering platform)
SNPs	single nucleotide polymorphisms
VFDB	Virulence Factor Database
VIBRANT	Virus Identification By iteRative ANnoTation
VIRIDIC	Virus Intergenomic Distance Calculator
WGS	whole-genome sequencing
WTA	wall teichoic acids
DNA	deoxyribonucleic acid
